# Idiopathic isolated clitoromegaly: A report of two cases

**DOI:** 10.1186/1742-4755-1-4

**Published:** 2004-10-04

**Authors:** Eray Copcu, Alper Aktas, Nazan Sivrioglu, Ozgen Copcu, Yucel Oztan

**Affiliations:** 1 Plastic and Reconstructive Surgery Department, Medical Faculty, Adnan Menderes University, Aydin, TURKEY; 2 Plastic and Reconstructive Surgery Department, Samsun State Hospital, Samsun, Turkey; 3 Anesthesiology and Reanimation Department, Aydin State Hospital, Aydin, Turkey; 4 Plastic and Reconstructive Surgery Department, Ataturk Training and Research Hospital, Izmir, TURKEY

## Abstract

**Background:**

Clitoromegaly is a frequent congenital malformation, but acquired clitoral enlargement is relatively rare.

**Methods:**

Two acquired clitoromegaly cases treated in Atatürk Training Hospital, Izmir, Turkey are presented.

**Results:**

History from both patients revealed clitoromegaly over the last three years. Neither gynecological nor systemic abnormalities were detected in either patient. Karyotype analyses and hormonal tests were normal. Abdominal and gynaecological ultrasound did not show any cystic lesion or other abnormal finding. Computerized tomography scan of the adrenal glands was normal. Clitoroplasty with preservation of neurovascular pedicles was performed for the treatment of clitoromegaly.

**Conclusion:**

The patients were diagnosed as "idiopathic isolated" clitoromegaly. To the best of our knowledge, there has been no detailed report about idiopathic clitoromegaly in the literature.

## Case reports

Two cases with clitoromegaly were treated in Atatürk Training Hospital, Izmir, Turkey.

**Figure 1:**
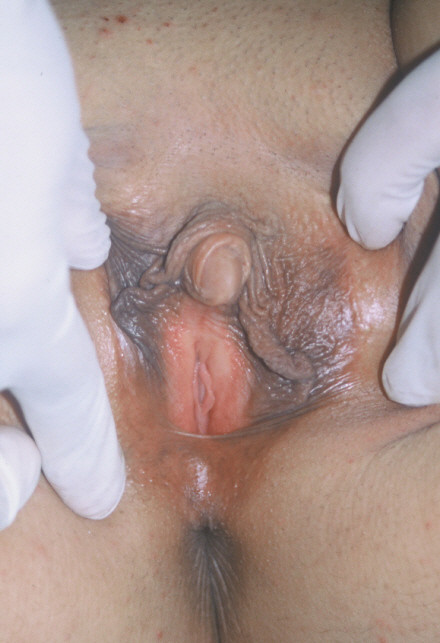
View of the Case 1.

**Figure 2:**
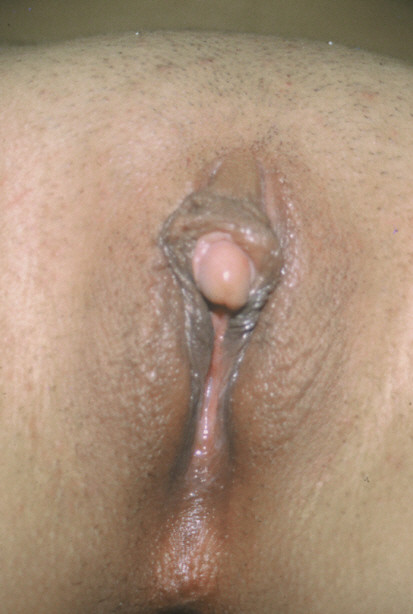
View of the Case 2.

A 22-year-old gravida 0 (case 1) and 19-year-old gravida 0 (case 2) presented with acquired clitoromegaly, leading to psychological distress. Histories taken from both patients revealed a gradually growing clitoris in the last three years, no history of drug abuse or family history of clitoromegaly and no clitoral irritation secondary to masturbation or other sexual functions. Case one had a phallus 20 mm in length that increased to 30 mm on arousal (Figure 1) and case two had a phallus 30 mm in length, which increased to 40 mm with arousal (Figure 2). Secondary sexual features were normal in both cases. Sexual hair was normal and there were no signs of hirsutism. The patients were not obese, weighing 65 and 68 kg, respectively. Neither patient had any sign of polycystic ovaries. No gynaecological or systemic abnormalities were detected in either patient. The only clinical finding was 'isolated clitoromegaly' on physical examination.

Karyotype analysis was done in both cases and reported as 46, XX. Results of routine laboratory tests were normal. In addition, serum electrolytes, oestradiol, sex hormone binding globulin (SHBG), testosterone, androstenedione, dehydroepiandrosterone sulphate (DHEA-S), follicule stimulating hormone (FSH), luteinizing hormone (LH), 17 hydroxy progesterone (17-OH-P), prolactin, adrenocorticotropic hormone (ACTH), cortisol, placental lactogen (PL), deoxycorticosterone, deoxycortisol 11, triiodothyronine (T3), thyroxine (T4), thyroid stimulating hormone (TSH), beta-human chorionic gonadotrophin (β-hCG), carcinoembryonic antigen (CEA) were measured before the operations and the results were normal. 17-ketosteroid output in 24-hour-urine specimen was normal in both patients. Abdominal and gynecological ultrasound did not show any cystic lesion or abnormal finding. Computed tomography scan of the adrenal glands was normal.

No abnormality suggestive of a possible relation to clitoromegaly was found in all laboratory and radiological tests.

**Figure 3:**
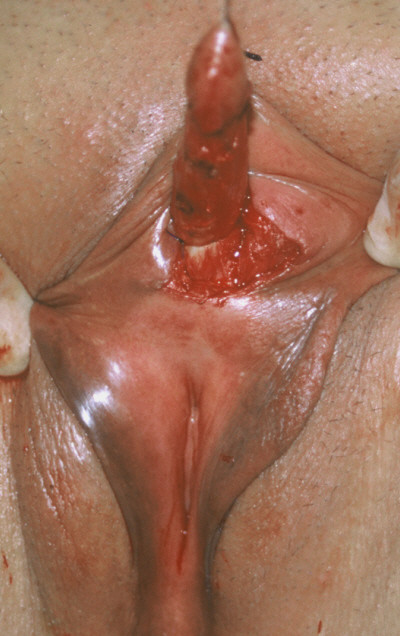
Traction of the clitoris per-operatively.

Both patients underwent clitoroplasty with preservation of the neurovascular pedicles under general anesthesia. A traction suture of 3/0 nylon was placed in the glans of clitoris (Figure 3). An incision was made on the lateral phallus perpendicular to the axis of the clitoral shaft, and carried through a 270 degree semicircular arc to the base of the glans as described by Papageorgoiou et al [1]. Two longitudinal incisions were made lateral to the dorsal neurovascular bundle. Two crura were identified, clamped and the mid-body of the clitoris was resected. The base of the glans was sutured to the divided corpora with 4/0 vicryl, and proximal and distal ends of the corpora were closed with 4/0 vicryl. The skin was closed with 4/0 vicryl sutures as well. Histopathological examinations of the resected specimens showed "normal corporal tissue". There was no abnormal finding on microscopic examination of the specimen obtained from clitoral and submucosal tissue.

Patients were followed up for one year after the operation. There was no early or late post-operative complication. Sensation was normal and patients were satisfied with the aesthetical and functional results.

## Discussion

Clitoromegaly is a frequently seen congenital malformation, but acquired clitoral enlargement is rarely detected [2]. A detailed history and physical examination are required for the evaluation of clitoral enlargement because clitoromegaly may result from a variety of conditions [3]. The causes of clitoromegaly can be classified into four groups; hormonal conditions, non-hormonal conditions, pseudoclitoromegaly and idiopathic clitoromegaly (Table 1).

**Table 1:** Classification of the clitoromegaly based on causative factors

Causative factors of clitoromegaly
A. **Hormonal conditions**
1. Endocrinopathies
2. Masculinizing tumors
3. Exposure to the androgens
4. Syndromes
B. **Non-Hormonal conditions**
1. Neurofibromatosis
2. Epidermoid cysts
3. Syndromes
4. Nevus
C. **Pseudoclitoromegaly**
D. **Idiopathic**

Endocrinopathies, masculinizing tumors, exposure to the androgens and various syndromes are the main hormonal causes of clitoromegaly. The most common cause is female pseudohermaphroditism secondary to congenital adrenal hyperplasia (CAH) or adrenogenital syndrome, caused by an enzyme defect in the normal pathway of steroid biosynthesis [4]. Virilization of the external genitalia may cause profound clitoromegaly but rarely causes formation of a true penile urethra. However, clitoromegaly may be accompanied by fusion of the labioscrotal folds and perineoscrotal hypospadias, and a persistence of the urogenital sinus closing the external opening of the vagina [5].

Bilateral hilus cell tumors of the ovary, steroid producing gonadal tumors, adrenal androgen-secreting carcinoma, Leydig cell tumor of the ovaries and metastatic carcinosarcoma of the urinary bladder have been reported to cause clitoromegaly [6-9].

Fetal exposure to danazol has been described as cause for clitoromegaly [10]. An interesting case was reported by Akcam and Topaloglu of clitoromegaly possibly following blood transfusion from an adult in a premature infant [11].

Among the non-hormonal conditions are neurofibromatosis (NF)[12], epidermoid cysts[3], various syndromes and nevus lipomatous cutaneous superficialis. The majority of clitoromegaly cases related to NF are congenital.

Clitoral cysts arise from epidermis displaced into the dermis or the subcutaneous tissue either during the prenatal period or after a trauma.

Various syndromes resulting from non-hormonal conditions may cause clitoromegaly. Kazlauskaite et al reported a case diagnosed as congenital generalized lipodystrophy (CGL) presenting with generalized body-fat loss, prominent musculature, hepatomegaly, clitoromegaly and mild hirsutism [13]. CGL is an autosomal recessive disorder characterized by severe metabolic derangement associated with the absence of subcutaneous adipose tissue and clitoromegaly. Turner syndrome (TS) is a chromosomal disorder in females and results from a partial or complete loss of an X chromosome. Abnormalities include short stature and gonadal dysgenesis. Haddad et al presented a case of clitoromegaly and TS [15]. Fraser syndrome is another rare cause of clitoromegaly [14].

Androgen insensitivity syndrome is a heterogeneous disorder with a wide spectrum of phenotypic abnormalities, ranging from a complete female phenotype to ambiguous forms that more closely resemble males. The primary abnormality is a defective androgen receptor protein due to mutation of the androgen receptor gene.

Nevus lipomatous cutaneous superficialis (NLCS) is a relatively rare condition characterized by groups of ectopic fat cells dispersed in various parts of the body [16] that may cause clitoromegaly when located on the clitoris.

Pseudohypertrophy of the clitoris has been reported in small girls due to masturbation: manipulations of the skin of prepuce leads to repeated mechanical trauma, which expands the prepuce and labia minora, thus imitating true clitoral enlargement [2].

The objectives of clitoroplasty are preservation of sexual arousal function and sensation, and cosmetic. Historically, until 1960s, clitoral hypertrophy was treated surgically by amputation (clitoridectomy)[4]. Surgical methods for correction of clitoral hypertrophy were first described in 1934 by Young, who performed an operation for clitoral reduction in a child with CAH [17]. Several clitoroplasty methods have been reported, but few describe preservation of dorsal and ventral neurovascular bundles in sexually mature women. Clitoroplasty with preservation of the neurovascular pedicle may be the optimal operative technique for the treatment of clitoromegaly.

## Competing interests

The authors declare that they have no competing interests.

## Authors' contributions

**EC **conceived the study and prepared the manuscript draft for submission. **AA, NS, OC **and **YO **did the literature search and participated in the preparation of the manuscript.

All authors read and approved the final manuscript.

## References

[B1] Papageorgiou T, Hearns-Stokes R, Peppas D, Segars JH (2000). Clitoroplasty with preservation of neurovascular pedicles. Obstet Gynecol.

[B2] Horejsi J (1997). Acquired clitoral enlargement. Diagnosis and treatment. Ann N Y Acad Sci.

[B3] Linck D, Hayes MF (2002). Clitoral cyst as a cause of ambiguous genitalia. Obstet Gynecol.

[B4] Bellinger MF, Ehrlich RM, Alter GJ (1999). Feminizing genitoplasty and vaginoplasty. Reconstructive and Plastic Surgery of the External Genitelia.

[B5] Wilson JD (2003). Formation of sexual phenotypes. The Endocrinologist.

[B6] Baramki TA, Leddy AL, Woodruff JD (1983). Bilateral hilus cell tumors of the ovary. Obstet Gynecol.

[B7] Castelazo-Ayala L, Zarate A, Mac Gregor C, Soria J, Dominguez O (1971). Steroid production by gonadal tumors in male pseudo-hermaphroditism with isolated clitoromegaly. Biochemical studies in vivo. Steroidologia.

[B8] Falsetti L, Salinaro F, Chiaramonte M (1995). Adrenal androgen-secreting carcinoma in a fertile woman. Acta Eur Fertil.

[B9] Ichinohasama R, Teshima S, Kishi K, Mukai K, Tsunematsu R, Ishii-Ohba H, Shimosato Y (1989). Leydig cell tumor of the ovary associated with endometrial carcinoma and containing 17 beta-hydroxysteroid dehydrogenase. Int J Gynecol Pathol.

[B10] Brunskill PJ (1992). The effects of fetal exposure to danazol. Br J Obstet Gynaecol.

[B11] Akcam M, Topaloglu A (2003). Extremely immature infant who developed clitoromegaly during the second month of her postnatal life probably due to frequent whole blood transfusion from an adult male. Pediatr Int.

[B12] Yuksel H, Odabasi AR, Kafkas S, Onur E, Turgut M (2003). Clitoromegaly in type 2 neurofibromatosis: a case report and review of the literature. Eur J Gynaecol Oncol.

[B13] Kazlauskaite R, Santomauro AT, Goldman J, Silver K, Snitker S, Beamer BA, Yen CJ, Shuldiner AR, Wajchenberg BL (2001). A case of congenital generalized lipodystrophy: metabolic effects of four dietary regimens. Lack of association of CGL with polymorphism in the lamin A/C Gene. Clin Endocrinol (Oxf).

[B14] Chattopadhyay A, Kher AS, Udwadia AD, Sharma SV, Bharucha BA, Nicholson AD (1993). Fraser syndrome. J Postgrad Med.

[B15] Haddad NG, Vance GH, Eugster EA, Davis MM, Kaefer M (2003). Turner syndrome (45x) with clitoromegaly. J Urol.

[B16] Hattori R, Kubo T, Yano K, Tanemura A, Yamaguchi Y, Itami S, Hosokawa K (2003). Nevus lipomatosus cutaneous superficialis of the clitoris. Dermatol Surg.

[B17] Young HH (1937). Genital abnormalities, hermaphroditism and related adrenal disease.

